# 

**Published:** 1889-07

**Authors:** 


### TABLE OF CONTENTS.

ORIGINAL AND SELECTED.

Dysentery, by A. Jacobi ........ 241
Typho-malarial fever ........ 246
Ergot in obstetric practice ........ 248
Bichloride of mercury in anemia ........ 250
Is apomorphine a safe emetic? ........ 251
Is acute rheumatism an inflammatory disease? .. 253
Electrolysis for the removal of superfluous hairs 254

ABSTRACTS &c.

On the use of hypnotics, &c. ........ 256
A growing science ........ 257
London Lancet says; Cocaine in extraction of teeth ........ 258
Prolapse of the uterus; Ammonium chloride for quick relief from drunkenness ........ 259
Infection of an infant through the milk of a tuberculous nurse; Ignipuncture of tonsils .... 260
New objection to the use of chloroform for anæsthesia; New antipyretic, pyrodin ........ 261
Tænia; Dr. Stewart states ........ 262
Electricity for cancer: Salts of phosphorus ...... 263
Treat. of croup; Cured by thermometer; Remedy for neuralgia ........ 264
Notes on the use of salicylates ........ 265
Treat. hypertrophy of tonsils ........ 266
Lanolin ........ 267
Progress in otology; Uralium: new hypnotic ... 268
How to clean hypodermic syringes ........ 269

SCIENTIFIC.

Electric watches and clocks; Automatic candles; A new antidote for morphine ........ 269
Toxity of the blood of salt water eels; Origin of the weather ........ 270

NOTES AND FORMULÆ.

Thompson's fever syrup; Ointment for piles; Diphtheria; Orchitis ........ 271

Tapeworm; For diarrhœa of children; Antiemetic; Gray oil; Diphtheria ........ 272

EDITORIAL.

Census returns-Physicians' registers; Wm. R. Warner & Co; Antipyrin; Pasteur and hydrophobia; A fat fee; Plucky ........ 273
Monobromated camphor; Physician's leisure library; Reed & Carnrick's preparation ...... 274
Lion Brewery; Johnson's Cyclopedia; Upjohn Pill and Granule Co.; Tulane University; I. Phillips; Southern Medical College advertisement; The senses; American resorts ........ 275
Insane in foreign countries; Suggestive therapeutics ........ 276
Fee bill; Prevention of yellow fever; Pamphlets received ........ 277
Word and Works; Seventh Decennial Convention; Dr. D. Gann writes; Receipted for 1889 ........ 278
Special notes ........ 279-280

